# Prenatal Alcohol Exposure and Transient Systemic Hypoxia–Ischemia Result in Subtle Alterations in Dendritic Complexity in Medial Frontal Cortical Neurons in Juvenile and Young Adult Rat Offspring in a Pilot Study

**DOI:** 10.3390/cells13231983

**Published:** 2024-11-30

**Authors:** Zarena M. Dominguez, Suzy Davies, Nathaniel G. Pavlik, Jessie C. Newville, Brooke R. Hafer, Clement P. Jose, Jessica Gross, Roberto N. Almeida Mancero, Lauren L. Jantzie, Daniel D. Savage, Jessie R. Maxwell

**Affiliations:** 1 Department of Pediatrics, University of New Mexico School of Medicine, Albuquerque, NM 87131, USA; zmdominguez@salud.unm.edu (Z.M.D.); dsavage@salud.unm.edu (D.D.S.); 2 Department of Neurosciences, University of New Mexico Health Sciences Center, Albuquerque, NM 87106, USA; sudavies@salud.unm.edu (S.D.); brdunn@salud.unm.edu (B.R.H.);; 3 School of Medicine, University of New Mexico Health Sciences Center, Albuquerque, NM 87106, USA; cpjose@salud.unm.edu; 4 Clinical and Translational Science Center, University of New Mexico, Albuquerque, NM 87106, USA; jmgross@salud.unm.edu; 5 Division of Neonatal-Perinatal Medicine, Department of Pediatrics, Johns Hopkins University School of Medicine, Baltimore, MD 21205, USA; ljantzie@jhmi.edu; 6 Division of Pediatric Neurosurgery, Department of Neurosurgery, Johns Hopkins University School of Medicine, Baltimore, MD 21205, USA; 7 Department of Neurology, Johns Hopkins University School of Medicine, Baltimore, MD 21205, USA; 8 Department of Neurology, Kennedy Krieger Institute, Baltimore, MD 21205, USA

**Keywords:** prenatal alcohol exposure, placental insufficiency, pyramidal neuron, prefrontal cortex, dendrites

## Abstract

Prenatal alcohol exposure (PAE) is associated with long-term neurodevelopmental deficits resulting in impaired executive functioning and motor control. Intriguingly, PAE has been linked with an increased risk of transient systemic hypoxia–ischemia (TSHI), which alone results in suboptimal fetal growth and neurodevelopmental consequences. Here, using two translationally relevant preclinical models, we investigated the short-term and lasting effects of PAE and TSHI on the morphology of the medial prefrontal cortex (mPFC), a region important in executive function, and tested whether PAE interacts with TSHI to produce a distinct pattern of injury relative to either condition alone. The four experimental groups included sham (saccharin water, no TSHI), PAE (5% alcohol, no TSHI), TSHI (saccharin water, TSHI), and PAE+TSHI (5% alcohol, TSHI). Brains were extracted for Golgi–Cox staining at Postnatal Day 35 (P35) or P100 and processed for 3D Sholl analysis. The analysis of the mPFC at P35 showed no significant differences in the number of branches or dendritic length overall, although the impact of TSHI compared to alcohol was significant for both. There were no significant differences in the number of Sholl intersections overall at P35, although a sex difference was noted in PAE offspring. At P100, analysis of filament dendritic length and branching number was also significantly impacted by TSHI compared to alcohol. Interestingly, sex was also a significant factor when assessing the impact of alcohol. PAE and TSHI both had an insignificantly increased number of Sholl intersections at P100 compared to the control. The observed changes to dendritic complexity at P100 demonstrate altered neuronal morphology in the mPFC that endure into adulthood. Given the importance of the mPFC in executive functioning, these pilot data provide insight into morphological changes that may contribute to the neurobehavioral deficits observed following exposure to PAE and TSHI and highlight the need for additional investigations into this area.

## 1. Introduction

Globally, alcohol consumption by pregnant women is common, resulting in an alarming number of infants exposed to alcohol during gestation [1,2]. Estimates of alcohol use during pregnancy vary in different countries, with Ireland, Australia, New Zealand, and the United Kingdom estimating up to 80% of pregnant individuals consuming alcohol [3]. In the United States, the prevalence of alcohol during pregnancy is estimated at around 10% [3], with nearly 7% of individuals reducing their alcohol intake during pregnancy [4]. Prenatal alcohol exposure (PAE) can disrupt the complex and interdependent steps in fetal neurodevelopment, producing an array of acute and chronic cognitive, behavioral, and motor deficits, collectively referred to as fetal alcohol spectrum disorders (FASD). Recent studies using a prospective approach estimate that the prevalence of FASD in school-aged children in the United States is as high as 9% [5,6,7,8], making PAE the leading cause of birth defects and intellectual disability [9]. A neuropsychological hallmark of children with FASD is difficulty in processing complex information, requiring greater involvement of brain regions responsible for executive functioning [10,11]. Notably, individuals with FASD have multiple compounding neurodevelopmental deficits that endure into adulthood [10], with an increased incidence of psychiatric disorders [12,13]. These deficits precipitate substantial challenges throughout the lifespan of those affected and highlight FASD as an important public health issue. Previous studies have demonstrated PAE-associated changes in the neuronal organization of the frontal cortex in adolescent rats that can affect their performance on behavioral tasks dependent on the functions and structural layout of the prefrontal cortex [14]. These alterations in neuronal organization may lead to impaired spatial response-dependent learning and indicate that dendritic complexity can impact executive function.

The executive and cognitive functions, often referred to as higher-level functions, involve brain regions such as the medial prefrontal cortex (mPFC) and hippocampus [15]. The mPFC is widely responsible for the ability to execute these different functions, including sensory neocortical systems, memory, attention, motor skills, judgment, and any such problem-solving or response inhibition skills [10,16,17,18]. The mPFC is extremely susceptible to alcohol exposure during development throughout pregnancy [16,19], which may partially explain some of the clinical alterations observed in FASD.

There has been considerable effort in determining other factors contributing to poor neurodevelopmental outcomes in the context of PAE. Changes in placental function have been observed in the setting of PAE [20,21,22,23]. Indeed, pregnant women who consume five or more alcoholic drinks per week have a two-fold increased risk of placental abnormalities, including poor placental perfusion [20,21,22,23]. These clinical findings have been replicated in preclinical models of prenatal alcohol exposure demonstrating impaired placentation [24] and reduced blood flow to the placenta in alcohol-exposed rat dams [25,26]. Furthermore, decreased placental weight, smaller placenta-to-birth weight ratio, and irregular placental protein expression have all been described in PAE [27,28,29,30]. Transient systemic hypoxia–ischemia (TSHI) alone significantly elevates the risk of growth failure, prematurity, and neurodevelopmental abnormalities, producing serious acute and chronic neurological and physical health consequences [31,32]. In the setting of PAE, the detrimental effects of TSHI on multiple fetal organ systems, particularly the brain, could be amplified. As noted by Kable et al., children with PAE and hemodynamic changes had worsening executive functioning [33].

Given that abnormalities in the development of the prefrontal cortex can result in neurological and psychiatric disorders [34,35,36,37], it is imperative to further characterize prenatal insults to better understand their impact(s) on long-term outcomes. Although both PAE and TSHI are common clinical entities [38,39], gaps in knowledge on the cumulative impact of PAE and TSHI on neurodevelopment remain. With each individual insult occurring frequently, it is critical to examine the impact these insults have together as the prevalence of co-occurrence is likely more common than has been recognized. In the current study, we utilized a well-established rat model of moderate PAE [40] in combination with a clinically relevant rat model of acute placental insufficiency induced by transient systemic hypoxia–ischemia [19,41,42,43,44,45,46,47,48]. Other studies have shown that the insult of alcohol treatment has resulted in significantly increased dendritic length and number of spines [49]. Similarly, studies of hypoxia–ischemia have shown increased dendritic complexity, which included increases in total dendritic length and the number of dendritic branches [50]. Therefore, we hypothesized that the combination of PAE and TSHI would result in greater increases in dendritic length and/or arborization within the mPFC compared to changes after either insult alone.

## 2. Materials and Methods

**Figure 1:**
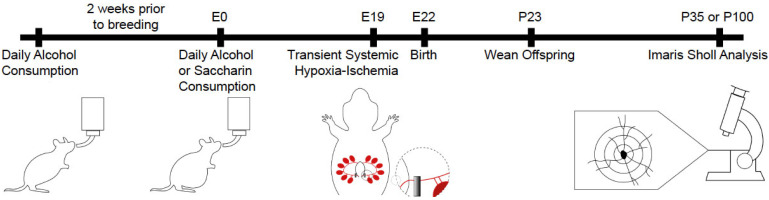
The overall experimental design. Rat dams voluntarily drank 0% alcohol for the first two days, 2.5% alcohol on days 3 and 4, and then 5% alcohol thereafter, for 4 h daily for 2 weeks prior to breeding to ensure intake was within expected levels. Rat dams consuming within 1 standard deviation of expected intake were then randomized to consume 5% alcohol or saccharin water during pregnancy. Prenatal alcohol exposure (PAE) occurred from Embryonic Day (E)0 to E18, whereby rat dams drank 5% alcohol or saccharin water for 4 h daily. Transient systemic hypoxia–ischemia (TSHI) or a sham procedure was performed on E19. Births occurred normally on E22 and pups were weaned on Postnatal Day (P)23. On P35 or P100, brain tissue was collected from offspring and stained with Golgi–Cox. Microscopy z-stack images were obtained and Imaris software was used to create a 3-dimensional rendering of the pyramidal neurons in the region of interest, with a Sholl analysis of dendritic complexity conducted.

All experimental procedures were approved by the Institutional Animal Care and Use Committee (IACUC) of the University of New Mexico Health Sciences Center, approval number 21-201201-HSC. All animal studies were carried out with standards of care and housing in accordance with the National Institutes of Health Guide for the Care and Use of Laboratory Animals, US Department of Health and Human Services. Long–Evans rats were obtained from Envigo Corporation (Indianapolis, IN, USA) and acclimated to the Animal Resource Facility environment for at least one week prior to any procedures, singly housed in plastic cages and maintained on a reverse 12 h dark, 12 h light schedule (lights off between 0900 and 2100). The rodent diet and tap water were available ad libitum. Offspring were group-housed until experimental endpoints were reached. The overall research design is presented in Figure 1.

### 2.1. Prenatal Alcohol Exposure (PAE)

Female Long–Evans rats, approximately 2.5 months of age, underwent a well-established moderate drinking paradigm as detailed by Davies et al. [40,51]. A moderate drinking paradigm was chosen as a majority of the human pregnant population falls within a mild to moderate use pattern [3,52]. The rats were first acclimated to drinking 5% alcohol in 0.066% saccharin water for 4 h daily (1000 to 1400 h). On drinking days 1 and 2, the saccharin water contained 0% alcohol, which was increased to 2.5% alcohol on days 3 and 4. From day 5 onward, the saccharin water contained 5% alcohol. Following a 2-week period of daily 5% alcohol consumption, only those rats that drank within one standard deviation of the average were continued in the experiment. Females were then randomly assigned to either a 0% alcohol (saccharin only) or a 5% alcohol drinking group (PAE) and paired with a proven male breeder until pregnancy was confirmed via the presence of a vaginal plug. Pregnant rat dams drank 5% alcohol or saccharin water from Embryonic Day (E)1 to E18 (from 1000 to 1400 daily), mimicking moderate PAE. Alcohol exposure across this time frame is the neurodevelopmental equivalent to the human first and second trimesters of brain development [41,53]. The volume of alcohol each rat dam drank was recorded, and the consumption of grams of alcohol per kilogram of body weight per day was calculated. This level of alcohol consumption produced blood alcohol concentrations (BACs) of 25.1 ± 3.3 mg/dL two hours after the introduction of the drinking tube on Gestational Day (GD)15, 16, or 17 [40]. This BAC value is roughly equivalent to a 120-pound woman consuming about 1 drink a day and is similar to other studies using this approach of voluntary drinking [40,54].

### 2.2. Transient Systemic Hypoxia–Ischemia (TSHI)

On E19, laparotomies were performed on one-half of the rat dams from both prenatal drinking groups to model an acute, or short-term, insult of in utero transient systemic hypoxia–ischemia (TSHI), as previously described by Jantzie et al. [19,41,42,43,44,45,46,47,48,55]. TSHI at this time is equivalent to a human late-second-trimester insult. Briefly, pregnant rat dams from both alcohol and saccharin treatment groups were anesthetized using 3% isoflurane, which was then lowered to 1% after all required incisions were made. Following exposure of the uterine horns via midline incision, two aneurysm clips were placed on the left and right uterine arteries. After sixty minutes, the clips were removed, the uterine horns were returned to the abdominal cavity, and the incisions sutured. This model represents a complete reperfusion, and thus, TSHI. Sham surgeries were performed, which included the open laparotomy with no uterine artery occlusion, on half of the rat dams from each prenatal drinking group. Post-operative rats were closely monitored until recovery.

### 2.3. Offspring Handling

On E22, offspring were delivered normally, and offspring weights were recorded from Postnatal Day (P)1 through P14, and on P21. On P23, pups were weaned and housed with littermates in sex-separate, standard housing not to exceed more than six offspring per cage. If there were more than six offspring of one sex, they were divided equally into two cages. Offspring had free feeding and tap water was available ad libitum. Enrichment (squiggles and tissue paper) was also available to the offspring.

### 2.4. Golgi Staining

Male and female P35 offspring (equivalent to a preadolescent human [56]) or P100 offspring (equivalent to an early adulthood human [57]) were deeply anesthetized with 50 mg/kg to 100 mg/kg of sodium pentobarbital (Vortech, Dearborn, MI, USA) and transcardially perfused with phosphate-buffered saline (PBS). Dissected brains were fixed in 4% paraformaldehyde (PFA) followed by 30% sucrose solution and then processed using Golgi–Cox staining methods and solutions included in the FD Rapid GolgiStain™ Kit (FD NeuroTechnologies, Inc., Colombia, MD, USA).

One-hundred-micron-thick coronal sections of Golgi-stained Layer 5 pyramidal neurons were generated using a Leica CM1950 cryostat from Bregma 3.20 mm–4.70 mm. Areas were defined using Paxinos and Watson’s *The Rat Brain in Stereotaxic Coordinates*, seventh edition [58]. Sections were mounted on gelatin-coated slides (FD NeuroTechnologies, Inc.), stained with Golgi–Cox, cover-slipped with Eukitt^®^ mounting medium (Sigma Aldrich, Inc., St. Louis, MO, USA), and stored in the dark at room temperature.

### 2.5. Confocal Microscopy and Sholl Analysis

**Figure 2:**
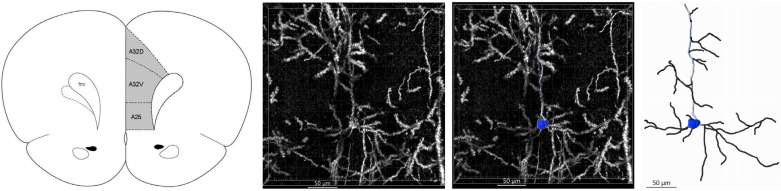
Isolation of dendritic arbors. After the z-stack imaging was obtained from the medial frontal cortex (noted on the left, shaded), the Imaris software was used to create 3D renderings. Pyramidal neurons were isolated with the “filaments function”, with the soma (blue) and apical dendrite (light gray) specifically noted.

Z-stacks of the mPFC were acquired using the reflective imaging function on a Leica TCS SP8 Confocal Microscope (Wetzlar, Germany) equipped with an Olympus 20× oil immersion objective (Tokyo, Japan). Equivalent microscope settings [format (µm): 1024 × 1024, line average: 5, laser: 488 nm, speed: 600, gain: 327, pinhole (au): 1.5, zoom: 1.89×, and z-step: 0.75 µm] were used throughout tissue imaging. The number of z-stack images obtained ranged from 20 to 35, with the goal of obtaining enough area to visualize an entire dendritic arbor. Using Imaris software v.10.1.0 (Oxford Instruments, Abingdon, UK), three-dimensional (3D) renderings of Layer 5 pyramidal neurons were created utilizing the “filaments function” (per Imaris software), and a three-dimensional Sholl analysis was conducted on six neurons, three in the left hemisphere and three in the right hemisphere, in the mPFC per animal (Figure 2 shows an overview of the neuron isolation process). Neurons for the mPFC region were selected at random. A 3D Sholl analysis quantifies dendritic complexity by counting the number of dendritic filaments that intersect spheres centered on the soma with radii increasing at ten-micron increments, as well as providing the dendritic length measurement. Neurons were selected for Sholl quantification based on their positions within the regions of interest to ensure their full dendritic arbors were visible; neurons whose processes extended beyond the region of interest were excluded. Intersections were measured at 10 µm increments. In this study, proximal refers to distances within 100 µm from the soma while those beyond 100 µm are considered distal [59]. Z-stacks previously obtained were opened in the surpass view and the “filaments” function was selected. Statistical data were collected after completion of tracing through Imaris using the “stats” function to collect the number of Sholl intersections overall. The dendritic length and number of branches were obtained from the “stats” function output. The imaging and analysis were completed by an individual blinded to the treatment conditions.

### 2.6. Statistics

The characteristics of the rat dams and offspring were analyzed. A Student’s *t*-test was used to assess differences in alcohol consumption between the PAE and PAE+TSHI groups. To assess differences among the four experimental groups, separate two-way analysis of variance (ANOVA) models with Tukey’s multiple comparisons test were conducted for each of the following dependent variables: (1) maternal weight gain during pregnancy, (2) litter size, (3) mortality rate, and (4) birth weight of the pups. Two three-way ANOVA models with Tukey’s multiple comparisons test were conducted to assess dendritic morphology. One model evaluated dendritic length as the dependent variable, while the other evaluated the number of branches. In both models, treatment group, sex, and either dendritic length or branch count were included as independent variables. To analyze the Sholl data, a repeated measures three-way ANOVA with Huynh–Feldt correction and Tukey’s multiple comparisons test was used. Sex, treatment group, and distance were included as independent variables for each measure collected. Statistical analyses were performed using IBM SPSS Statistics (v.29.0.2.0, Armonk, NY, USA) and GraphPad Prism 10 (v.10.2.3, Boston, MA, USA). Statistical significance was defined at *p* < 0.05.

## 3. Results

### 3.1. Dam and Offspring Characteristics

A summary of the maternal and offspring outcome data is provided in Table 1. At P35, three male and three female offspring from each of the four prenatal treatment groups (TSHI (n = 6; 3 male. 3 female), PAE (n = 6; 3 male, 3 female), PAE+TSHI (n = 6; 3 male, 3 female), and sham (n = 6; 3 male, 3 female)) were used, for a total of 24 offspring. P100 offspring across the four prenatal treatment groups (TSHI (n = 4; 2 male, 2 female), PAE (n = 4; 2 male, 2 female), PAE+TSHI (n = 6; 3 male, 3 female), and sham (n = 6; 3 male, 3 female)) were used for a total of 20 offspring. Thus, a total of 44 offspring and 31 litters were used. Rat dams consumed a daily average intake of alcohol of 2.17 ± 0.15 g/kg/day in the PAE group and 2.21 ± 0.07 in the PAE+TSHI group (Table 1), which did not differ significantly between the two groups (*p* = 0.77). The maternal weight gain during pregnancy did not differ between the four groups (F(3, 17) = 0.03; *p* = 0.99). Of note, one rat dam from the sham, TSHI, and PAE+TSHI groups and two rat dams from the PAE group did not have a weight obtained during week 3 of gestation; therefore, the weight change could not be calculated. The litter size was not significantly different (F(3, 20) = 0.70; *p* = 0.56) and offspring were delivered normally on E22. Although mortality was increased in placental insufficiency, this increase did not reach significance in this model nor in prior studies (F(3, 20) = 1.80; *p* = 0.18) [41,55]. No significant differences in litter weights were found between the control and the three treatment groups at birth or at P21 (F(3, 19) = 0.70; *p* = 0.55 and F(3, 20) = 0.10; *p* = 0.96, respectively).

### 3.2. Medial Frontal Cortex

Initial Sholl analysis was performed on six randomly selected pyramidal neurons within Layer 5 of the mPFC of each offspring: three from the left hemisphere and three from the right hemisphere. Analyses were completed to investigate the impact on specific subregions including the anterior cingulate, but no significant differences were observed. Figure 2 reviews the isolation process for dendritic arbors. A Student’s *t*-test revealed no significant differences between the left and right hemisphere values at P35 or P100. As a result, data from the two hemispheres were combined and averaged to yield a single value for each parameter per offspring.

### 3.3. Dendritic Length and Number of Branches

#### 3.3.1. P35 Analysis

**Figure 3:**
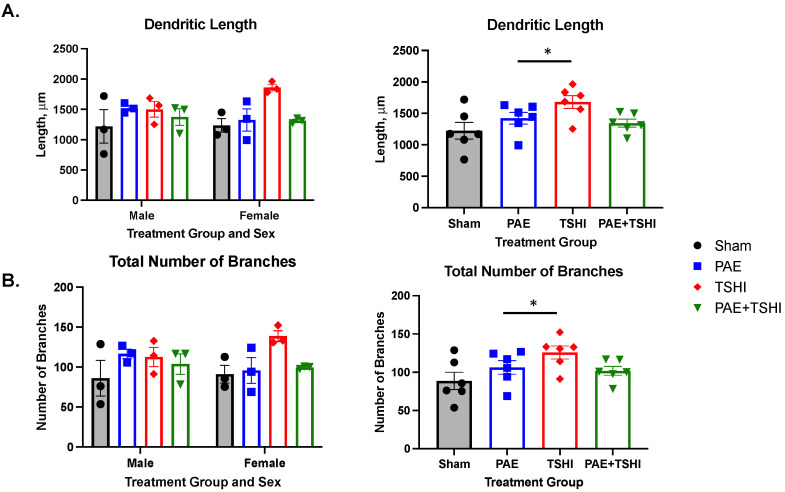
Dendritic morphology at P35. The total filament length was analyzed between treatment group and sex, and treatment group overall. The female TSHI group had the longest total dendritic length (**A**) as well as the highest amount of total number of branches (**B**). Tukey’s multiple comparison tests showed a difference between the TSHI and PAE groups for both dendritic length and the total number of branches. * *p* < 0.05.

The total filament dendritic length was not significantly different when accounting for sex and treatment group (F(3, 16) = 0.26; *p* = 0.62; see Figure 3A) with the three-way ANOVA model. Sex was not a significant contributor (F(1, 16) = 0.09, *p* = 0.77) with the multiple comparisons tests. The comparison of TSHI to PAE was significant (F(1, 16) = 6.95; *p* < 0.05). When analyzing the male-only data, the male sham group on average had the shortest total filament dendritic length (1218 µm), while the male PAE group had the longest total filament dendritic length (1521 µm); the PAE+TSHI male and male TSHI group values were in between at 1375 µm and 1501 µm, respectively. When analyzing the female-only data, the female average total filament dendritic length had a slightly different trend, with the TSHI group having the longest total filament dendritic length (1861 µm) and the sham group having the shortest total filament dendritic length (1235 µm). The PAE group had the second longest total filament dendritic length (1325 µm) with the PAE+TSHI group having the second shortest total filament dendritic length (1315 µm). No significant differences were observed in the male or female cohort.

The total number of branches was not significantly different when accounting for sex and treatment group (F(3, 16) = 0.02; *p* = 0.90; see Figure 3B) using the three-way ANOVA analysis. Sex was not a significant contributor independently (F(1, 16) = 0.03, *p* = 0.87) with the multiple comparisons tests. Interestingly, the comparison of TSHI to PAE was significant (F(1, 16) = 5.42; *p* < 0.05). Similar to the findings of the total filament dendritic length assessed based on sex, the total number of branches was highest in the male PAE group (117) and lowest in the male sham group (86); the male PAE+TSHI and male TSHI group values were in between at 104 and 113, respectively. No significant differences were observed in the male cohort. The total number of branches in the female cohort followed the trend from the female total filament dendritic length, with the TSHI group having the most branching (139), the sham group having the least branching (91), and the PAE and PAE+TSHI groups in the middle (96 and 100, respectively). No significant differences were observed in the female cohort.

#### 3.3.2. P100 Analysis

**Figure 4:**
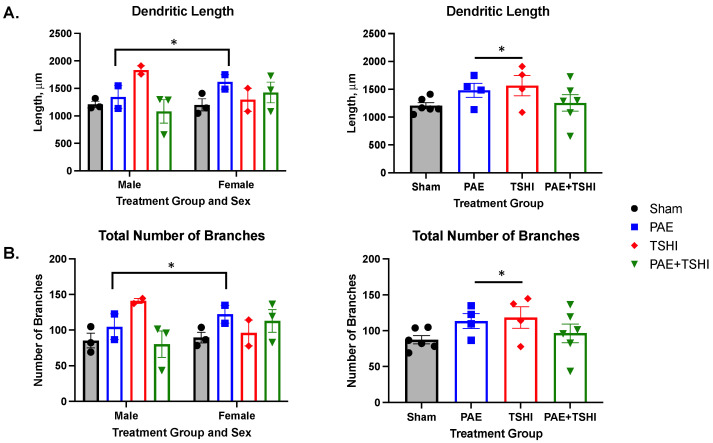
Dendritic morphology at P100. The total filament length was analyzed between treatment group and sex, and treatment group overall. The male TSHI group had the longest total dendritic length (**A**) as well as the highest amount of total number of branches (**B**). There was a significant difference between the PAE and TSHI groups in the dendritic length and the total number of branches. Sex had a significant impact on the dendritic length and the total number of branches for the PAE group. * *p* < 0.05.

We found no significant differences in total dendritic filament length across sex and treatment groups (F(3, 12) = 1.64; *p* = 0.23; see Figure 4A) with the three-way ANOVA analysis. The comparison of TSHI to PAE was significant (F(1, 12) = 6.25; *p* < 0.05) with multiple comparison tests. Additionally, sex was a significant factor for the comparison with PAE (F(1, 12) = 6.24; *p* < 0.05). In the male groups, the PAE+TSHI group had the shortest total dendritic filament length (1081 µm), while the TSHI group had the longest (1835 µm). The sham and PAE male groups showed intermediate lengths, measuring 1210 µm and 1343 µm, respectively. Tukey’s multiple comparisons test revealed a significant difference between the TSHI and PAE+TSHI male groups (*p* < 0.05). In the female groups, the pattern was somewhat different; the PAE group exhibited the longest total dendritic filament length (1618 µm) and the sham group had the shortest (1201 µm). The PAE+TSHI group had the second longest length (1425 µm), while the TSHI group had the second shortest (1294 µm). Tukey’s multiple comparisons test did not reveal any significant differences among the female groups.

The total number of branches did not differ significantly when we accounted for sex and treatment group (F(3, 12) = 2.4; *p* = 0.15; see Figure 4B) using the three-way ANOVA analysis. The comparison of TSHI to PAE was significant (F(1, 12) = 5.36; *p* < 0.05) with multiple comparison tests. Sex was again a significant factor for the comparison with PAE (F(1, 12) = 4.82; *p* < 0.05). In line with the findings for total filament dendritic length, the male TSHI group had the highest number of branches (141), while the male PAE+TSHI group had the lowest (80). The male sham and PAE groups showed intermediate levels of branching, with 85 and 105 branches, respectively. Tukey’s multiple comparisons test did not find any significant differences within the male cohort. In the female cohort, the branching pattern mirrored that of the total filament dendritic length, with the PAE group showing the highest number of branches (122) and the sham group the lowest (89). The TSHI and PAE+TSHI groups had intermediate branching, with 96 and 113 branches, respectively. Tukey’s multiple comparisons test did not reveal any significant differences among the female groups.

### 3.4. Sholl Analysis

#### 3.4.1. P35 Analysis

**Figure 5:**
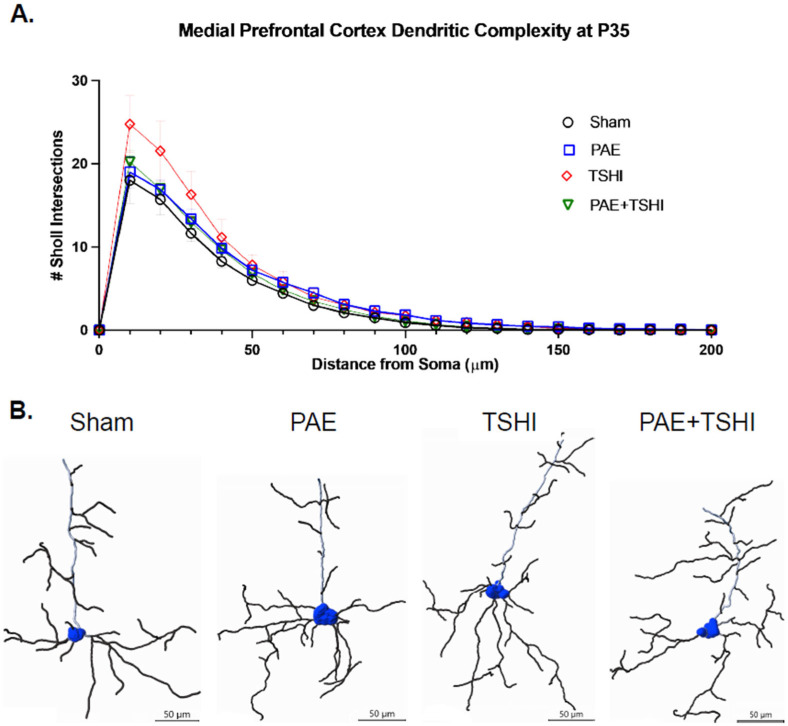
Sholl analysis in the medial frontal cortex at P35. The Sholl analysis was carried out in the medial frontal cortex region at P35. (**A**) Sholl analysis was completed on 6 pyramidal neurons per offspring with the number of intersections observed at each specific distance recorded per each treatment group. (**B**) Representative traces of neurons illustrate differences in branching complexity between treatment groups (scale bar = 50 μm). Although the TSHI group had longer dendritic length and more overall branching, there were no statistical differences found between the four treatment groups.

The three-way repeated measures ANOVA did not reveal statistical significance between the distance from the soma, sex of the offspring, and treatment group (F(7.29, 38.88) = 0.66; *p* = 0.71) with the number of intersections on the Sholl analysis (see Figure 5). As expected, distance alone was significant (F(2.43, 38.88) = 354.18; *p* < 0.001). There was no significant interaction observed between distance and sex (F(2.43, 38.88) = 0.24; *p* = 0.83). Interestingly, the interaction of distance and treatment group was trending towards significance; given the sample size, it is unclear whether there is a true lack of an interaction effect or if this is due to the sample size (F(7.29, 38.88) = 2.00; *p* = 0.08). Additionally, we evaluated the polynomial contrasts to further investigate the relationship between distance from the soma and dendritic branching. The tests of within-subject contrasts for distance alone showed polynomial contrasts and significance observed in linear (F(1, 16) = 539.36; *p* < 0.001), quadratic (F(1, 16) = 272.95; *p* < 0.001), cubic (F(1, 16) = 498.50; *p* < 0.001), and order 4-11 (F(1, 16) = 24.46-623.28; *p* < 0.001) and 13-15 (F(1, 16) = 4.82-8.54; *p* < 0.05) trends. These results reveal that the best-fit trend line is complex, with multiple curves, indicating that the overall trend of decreasing crossings is not consistent between distances. The distance, sex, and treatment group interaction showed significance in the polynomial contrasts of orders 17 (F(3, 16) = 4.84; *p* < 0.05) and 19 ((F3, 16) = 5.27; *p* = 0.01); see the Supplementary Materials, Table S1, for male data and Table S2 for female data.

Overall, while sex was not a significant factor (F(1, 16) = 0.02; *p* = 0.90), there were significant sex differences in the PAE group only. The PAE males and females were significantly different at 50 µm (*p* < 0.05; CI: 0.267 to 4.599) and 70 µm (*p* < 0.05; CI: 0.115 to 3.772). No other differences were noted between the sexes and the treatment group at any other distance. Additionally, a difference was observed within males but not within females. At 70 µm, the male sham and PAE groups were significantly different in the number of intersections present (*p* < 0.05; CI: −5.626 to −0.073). There were no other significant differences in male offspring between the sham, PAE, TSHI, and PAE+TSHI groups at any other distance from the soma. Within the female offspring, there were no significant differences between the sham, PAE, TSHI, and PAE+TSHI groups at any distance from the soma. No other distances had specific differences between groups.

#### 3.4.2. P100 Analysis

**Figure 6:**
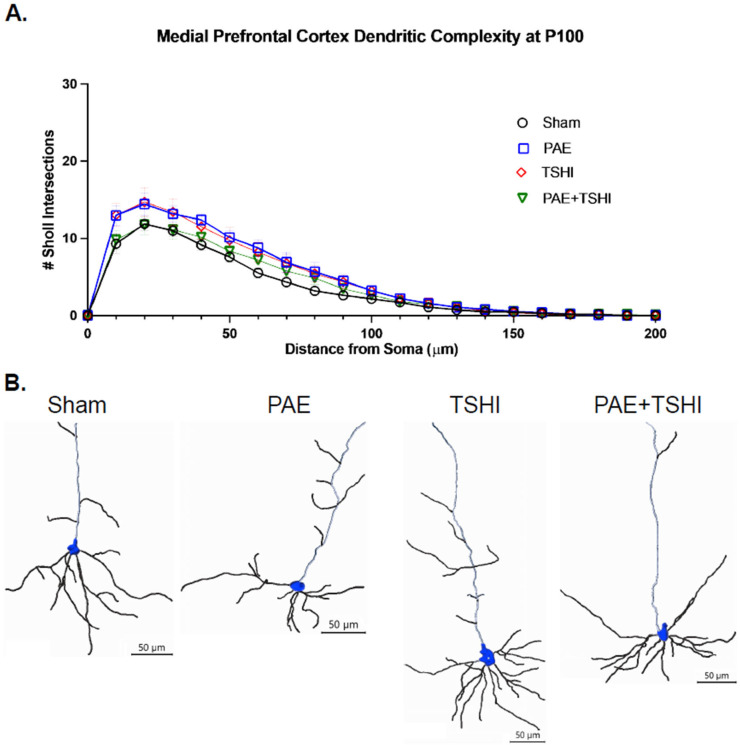
Sholl analysis in the medial frontal cortex at P100. The Sholl analysis was carried out in the medial frontal cortex (blue) region at P100. (**A**) Sholl analysis was completed on 6 pyramidal neurons per offspring with the number of intersections observed at each specific distance recorded per each treatment group. (**B**) Representative traces of neurons illustrate differences in branching complexity between treatment groups (scale bar = 50 μm). There were no statistical differences found between the four treatment groups.

We conducted a three-way repeated measures ANOVA, which did not find a statistically significant interaction between the distance from the soma, sex of the offspring, and treatment group with respect to the number of intersections in the Sholl analysis (F(11.44, 45.76) = 1.59; *p* = 0.13; see Figure 6). As expected, distance alone showed a significant effect (F(3.81, 45.76) = 245.34; *p* < 0.001). We did not observe a significant interaction between distance and sex (F(3.81, 45.76) = 0.31; *p* = 0.86).

To further explore the relationship between distance from the soma and dendritic branching, we evaluated polynomial contrasts. The tests of within-subject contrasts for distance alone showed significant polynomial trends: linear (F(1, 12) = 348.19; *p* < 0.001), cubic (F(1, 12) = 240.71; *p* < 0.001), and higher-order trends from order 4 to 8 (F(1, 12) = 23.48-364.15; *p* < 0.001), order 9 (F(1, 12) = 8.73; *p* = 0.01), and order 11 (F(1, 12) = 5.71; *p* = 0.03). These results again indicate that the best-fit trend line is complex and involves multiple curves, suggesting that the overall trend of decreasing crossings varies across different distances. Additionally, the interaction of distance, sex, and treatment group showed significance in order 14 (F(3, 12) = 3.71; *p* = 0.04). Sex was not a significant factor (F(1, 12) = 0.00; *p* = 0.99); see the Supplementary Materials, Table S3, for male data and Table S4 for female data.

## 4. Discussion

Given the profound long-term neurological deficits that PAE and TSHI produce and the region-specific effect these injuries have on dendritic development, a three-dimensional Sholl analysis was used to quantify dendritic complexity in layer V of the mPFC of juvenile and young adult rats. The data presented here represent pilot data, and the findings highlight the need for continued investigations in this area. The mPFC plays a significant role in cognitive and executive function processes, including attention and decision-making [17]. We hypothesized that PAE and TSHI would alter the dendritic complexity of the pyramidal neurons in this area, as previous work has established that mPFC morphology is vulnerable to developmental insult, with links to a multitude of neural developmental disorders [60]. The findings did not show significance overall, although TSHI and PAE did have small effects within multiple and pairwise comparisons. Additionally, sex differences were noted in the PAE group, which has been reported in other types of PAE studies [14,61,62,63,64,65].

The initial analysis showed no significant differences between sexes within the mPFC. While stress has been noted to result in sex-specific differences in dendritic spine complexity [66,67], not all prenatal alcohol exposure paradigms have reported sex as a significant factor [14,52,65,68]. Stress factors presented to pregnant rat dams have been observed to affect the changes in the complexity of the number of dendritic intersections, spine density, a shorter total dendritic length, and fewer mature spines in the mPFC [14,69]. Chronic stress decreases the length and branching of apical dendrites in the prefrontal cortex [14]. Stress has also been observed to influence spine density and dendritic tree structure in mPFC where there is decreased spine density and dendritic length [69]. The damage presented because of stress on the mPFC has been shown to be reversible in both humans and rats [14]. Therefore, it may be that the stressors in this paradigm were mild enough to not cause injury, or there could have been a resolution of that injury prior to our assessments at P35 and P100.

Any changes to pyramidal neuron morphology may have important consequences for mPFC function. Previous investigations have revealed that PAE significantly alters dendritic development and can result in reduced dendritic complexity in regions within the frontal lobe [70,71,72]. While overall our paradigm did not show significant differences, PAE was noted to have an impact. The multiple comparisons showed significance with PAE that was not noted with placental insufficiency, which may indicate that neurons in the mPFC are more sensitive to the alcohol insult than transient systemic hypoxia–ischemia. Structural changes may have profound impacts on neuronal function as dendritic profiles, such as dendrite diameter and distance from the soma, relate to the cell’s role in regional brain circuitry [70,71,72]. Spine number variations can lead to neural disorders such as autism spectrum disorder that can be associated with an impaired synaptic pruning process [69,73,74]. Additional studies are needed to determine if there are functional changes due to this impact, as hypothesized in conditions such as autism spectrum disorder.

The paradigm of PAE used here represents moderate exposure to alcohol during the first and second trimesters of gestation. In third-trimester rat models of binge drinking, the hippocampus and mPFC, both central to proper cognitive and executive functioning, are found to lose basilar dendritic complexity but have no effect on spine density [14,75,76]. Reduction in proximal basilar dendritic complexity could result in a lack of inhibitory signals from parvalbumin cells [14]. Apical dendrite exposure to alcohol found a significant decrease in spine density while there were no observed differences in dendritic complexity [14,69]. The differences in modeling (first- and second-trimester exposure compared to third-trimester exposure) may partially explain why our results differ from prior reports.

The transient systemic hypoxia–ischemia procedure was induced later in gestation. Transient systemic hypoxia–ischemia has also been shown to have varied effects on different types of neurons. In ovine models, a decrease in apical complexity and an increase in basal complexity is seen in the CA1 region of the hippocampus [50], while subplate neurons exhibit decreased basilar complexity [77]. Dendrite shortening can also be seen through the response of the placenta in vitro to hypoxia [74], and lack of long-term potentiation prevents further growth of dendritic spines [69]. If the TSHI increased overall complexity (such as the slight increase in Sholl intersections at P35 and increased dendritic length and branch number at both P35 and P100) and the PAE decreased overall complexity, these together may have resulted in an apparent “normalization” in the dendritic complexity. As noted at P100, the dendritic length and number of branches in the PAE+TSHI are very similar to those observed in the sham cohort. Similarly, the number of Sholl intersections is also very similar between the PAE+TSHI and sham cohorts.

We acknowledge that this study represents pilot results due to the limited sample size, and therefore continued studies are needed to further expand the sample size to allow for broader application of these results. Given that changes in dendritic arborization have been linked to disorders and functional changes, these data inspire questions for future studies including the effect of these and other morphological alternations on the behavior of TSHI, PAE, and PAE+TSHI rats into adulthood. We recognize that the mPFC is a relatively large brain region, and the location of the neurons analyzed could impact the results observed. We also recognize that our acute TSHI model could impact the results we observed since we did not observe any growth deficits. Therefore, other important regions and smaller subregions, such as the infralimbic or prelimbic cortex, should be analyzed in future studies to determine if these prenatal insults impact the dendritic arborization in areas critical to control of impulsivity, compulsivity, self-control, and stress [78,79,80]. Along with this, a chronic model of TSHI can help determine the impact of the prenatal insult on dendritic arborization within the mPFC and other important regions within the brain. Further, other imaging modalities, such as magnetic resonance imaging (MRI), could yield important information on the microstructural integrity and connectivity of these brain regions to aid our understanding of underlying mechanisms contributing to perinatal injury pathogenesis. Additionally, in vivo electrophysiology may be used to assess functionality in specific regions.

## 5. Conclusions

Alterations in the dendritic arborization, although not consistently significant, are observed following the insults of PAE and TSHI. Additional studies investigating the functional outcomes are needed to determine if the changes observed may result in altered function.

## Figures and Tables

**Table 1:** Dam and offspring characteristics. This table shows the rat dam and offspring characteristics. The daily average alcohol intake in the rat dams receiving 5% alcohol was calculated as grams of alcohol consumed per kilogram of body weight per day. Across the three-week gestational exposure period, PAE rat dams averaged a daily alcohol consumption of 2.21 ± 0.07 g/kg. The maternal weight gain through Embryonic Day 18 (E18) is shown for all rat dams, measured as the gram increase in body weight through E18, and was not significantly different between groups (*p* = 0.99). The average litter size is included for each experimental group, as well as the mortality rate [percentage of death based on fetal count during surgery compared to live pups on Postnatal Day 2 (P2)]. The birth weight of the offspring per litter and experimental group was obtained, as well as the weight at P21. Note that there was no significant difference in the maternal litter size, offspring birth weight, or offspring weight at P21 (all data are shown as the mean value ± standard error of the mean (SEM); n = 31 dams, n= 44 offspring).

	Sham	Transient Systemic Hypoxia-Ischemia	Prenatal Alcohol Exposure	Prenatal Alcohol Exposure + Transient Systemic Hypoxia-Ischemia	F Value	*p* Value
Overall daily four-hour alcohol consumption *	NA	NA	2.17 ± 0.15	2.21 ± 0.07		0.77
Week 1 alcohol consumption*	NA	NA	1.82 ± 0.25	2.09 ± 0.11		0.34
Week 2 alcohol consumption*	NA	NA	2.30 ± 0.13	2.23 ± 0.11		0.67
Week 3 alcohol consumption*	NA	NA	2.37 ± 0.14	2.33 ± 0.07		0.80
Maternal weight gain	87.86 ± 4.80	87.43 ± 2.50	91.60 ± 3.17	88.29 ± 3.03	0.03	0.99
Litter size (number of live pups/litter)	10.63 ± 0.89	9.50 ± 0.89	11.14 ± 0.67	10.38 ± 0.71	0.70	0.56
Mortality rate (% death from surgery to birth)	4.00 ± 3.12	17.00 ± 5.31	3.86 ± 3.86	13.13 ± 6.83	1.80	0.18
Offspring birth weight (grams)	6.54 ± 0.47	6.66 ± 0.26	6.39 ± 0.20	6.18 ± 0.19	0.70	0.55
Offspring weight on postnatal day 21 (grams)	43.32 ± 2.62	44.76 ± 2.88	43.27 ± 2.37	44.55 ± 1.86	0.10	0.96

* *p* value was calculated using a Student’s *t*-test, while the remainder of the *p* values were calculated with a two-way ANOVA with Tukey’s correction. *p* values and F values for two-way ANOVA calculations are shown for the findings between treatment groups [sham, transient systemic hypoxia–ischemia (TSHI), prenatal alcohol exposure (PAE), and transient systemic hypoxia–ischemia + prenatal alcohol exposure (PAE+TSHI)]. NA = not applicable as alcohol was not consumed.

## Data Availability

Datasets are available upon request; the raw data supporting the conclusions of this article will be made available by the authors, without undue reservation.

## Supplementary Materials

The following supporting information can be downloaded at https://www.mdpi.com/article/10.3390/cells13231983/s1.
