# Supplementary material for: Prenatal Alcohol Exposure and Transient Systemic Hypoxia–Ischemia Result in Subtle Alterations in Dendritic Complexity in Medial Frontal Cortical Neurons in Juvenile and Young Adult Rat Offspring in a Pilot Study
Source: Cells. 2024 Nov 30;13(23):1983. doi: 10.3390/cells13231983 (PMC11640287; doi:10.3390/cells13231983)
Supplement: Supplementary file 1 [file cells-13-01983-s001.zip › Figure S1.pdf]

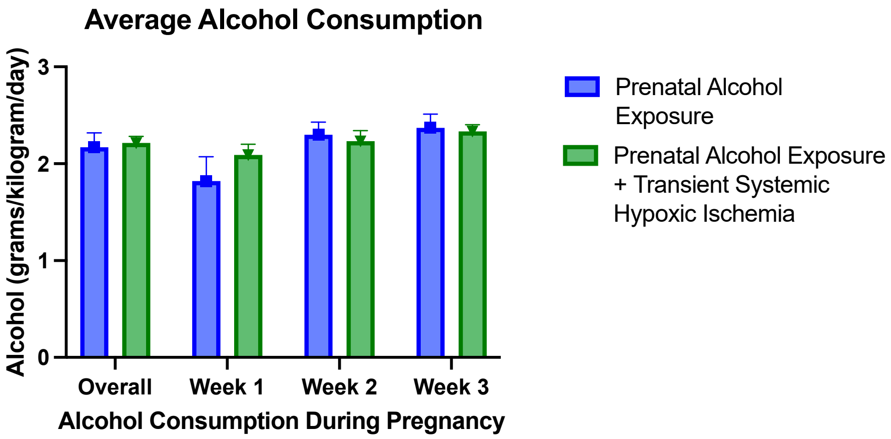

Supplementary Figure S1. A comparison of the alcohol consumption between the prenatal alcohol exposure (PAE) and the PAE with transient systemic hypoxia ischemia (TSHI) groups during pregnancy and by week is shown. There was no significant difference observed in the alcohol consumed between groups.
