# Supplementary material for: Prenatal Alcohol Exposure and Transient Systemic Hypoxia–Ischemia Result in Subtle Alterations in Dendritic Complexity in Medial Frontal Cortical Neurons in Juvenile and Young Adult Rat Offspring in a Pilot Study
Source: Cells. 2024 Nov 30;13(23):1983. doi: 10.3390/cells13231983 (PMC11640287; doi:10.3390/cells13231983)
Supplement: Supplementary file 1 [file cells-13-01983-s001.zip › Supplemental Tables.pdf]

Supplemental Table 1

Sex	Treatment Group	Distance	Mean	Standard Error	95% Confidence Interval	
					Lower Bound	Upper Bound
Male	Sham	1	0.00	0.00	0.00	0.00
		2	17.60	3.15	10.93	24.27
		3	15.55	2.12	11.06	20.04
		4	11.33	1.59	7.97	14.70
		5	7.95	1.17	5.47	10.42
		6	5.55	0.81	3.83	7.28
		7	4.39	0.67	2.96	5.81
		8	2.78	0.57	1.57	3.98
		9	2.06	0.42	1.16	2.95
		10	1.39	0.35	0.64	2.14
		11	5.33	1.73	1.67	9.00
		12	0.50	0.31	-0.15	1.15
		13	0.33	0.24	-0.19	0.85
		14	0.33	0.23	-0.16	0.82
		15	0.17	0.17	-0.19	0.52
		16	0.17	0.15	-0.15	0.48
		17	0.11	0.13	-0.17	0.39
		18	0.00	0.09	-0.20	0.20
		19	0.00	0.08	-0.16	0.16
		20	0.00	0.05	-0.11	0.11
		21	0.00	0.03	-0.06	0.06
	PAE	1	0.00	0.00	0.00	0.00
		2	17.89	3.15	11.22	24.56
		3	16.32	2.12	11.83	20.81
		4	13.78	1.59	10.42	17.14
		5	10.89	1.17	8.41	13.36
		6	7.89	0.81	6.17	9.61
		7	6.44	0.67	5.02	7.87
		8	5.05	0.57	3.85	6.26
		9	3.33	0.42	2.44	4.23
		10	2.72	0.35	1.97	3.47
		11	1.45	1.73	-2.22	5.11
		12	1.00	0.31	0.35	1.65
		13	0.72	0.24	0.21	1.24
		14	0.61	0.23	0.12	1.10
		15	0.44	0.17	0.09	0.80
		16	0.33	0.15	0.02	0.65
		17	0.22	0.13	-0.05	0.50
		18	0.11	0.09	-0.09	0.31
		19	0.11	0.08	-0.05	0.27
		20	0.06	0.05	-0.05	0.17
		21	0.00	0.03	-0.06	0.06
	PI	1	0.00	0.00	0.00	0.00
		2	22.88	3.15	16.21	29.55
		3	19.45	2.12	14.96	23.94
		4	14.78	1.59	11.41	18.14
		5	10.33	1.17	7.86	12.81
		6	7.44	0.81	5.72	9.16
		7	5.00	0.67	3.58	6.42
		8	3.78	0.57	2.57	4.98
		9	2.67	0.42	1.77	3.56
		10	2.05	0.35	1.30	2.80
		11	1.72	1.73	-1.94	5.39
		12	1.06	0.31	0.41	1.70
		13	0.89	0.24	0.37	1.40
		14	0.72	0.23	0.23	1.21
		15	0.45	0.17	0.09	0.80
		16	0.17	0.15	-0.15	0.48
		17	0.11	0.13	-0.17	0.39
		18	0.06	0.09	-0.14	0.25
		19	0.06	0.08	-0.11	0.22
		20	0.06	0.05	-0.05	0.17
		21	0.00	0.03	-0.06	0.06
	PAE+PI	1	0.00	0.00	0.00	0.00
		2	21.83	3.15	15.16	28.51
		3	18.06	2.12	13.57	22.55
		4	13.50	1.59	10.14	16.86
		5	10.00	1.17	7.53	12.47
		6	7.06	0.81	5.34	8.78
		7	4.11	0.67	2.69	5.53
		8	3.28	0.57	2.07	4.48
		9	2.39	0.42	1.49	3.28
		10	1.55	0.35	0.80	2.30
		11	0.89	1.73	-2.78	4.56
		12	0.61	0.31	-0.04	1.26
		13	0.39	0.24	-0.13	0.90
		14	0.17	0.23	-0.32	0.65
		15	0.11	0.17	-0.24	0.46
		16	0.06	0.15	-0.26	0.37
		17	0.06	0.13	-0.22	0.33
		18	0.00	0.09	-0.20	0.20
		19	0.00	0.08	-0.16	0.16
		20	0.00	0.05	-0.11	0.11
		21	0.00	0.03	-0.06	0.06

Supplemental Table 2

Sex	Treatment Group	Distance	Mean	Standard Error	95% Confidence Interval	
					Lower Bound	Upper Bound
Female	Sham	1	0.00	0.00	0.00	0.00
		2	18.43	3.15	11.76	25.11
		3	15.89	2.12	11.40	20.38
		4	11.95	1.59	8.58	15.31
		5	8.61	1.17	6.14	11.08
		6	6.44	0.81	4.72	8.17
		7	4.50	0.67	3.08	5.92
		8	3.17	0.57	1.96	4.37
		9	2.10	0.42	1.21	2.99
		10	1.55	0.35	0.79	2.30
		11	1.00	1.73	-2.67	4.66
		12	0.67	0.31	0.02	1.31
		13	0.28	0.24	-0.24	0.80
		14	0.11	0.23	-0.38	0.60
		15	0.00	0.17	-0.35	0.35
		16	0.00	0.15	-0.31	0.31
		17	0.00	0.13	-0.28	0.28
		18	0.00	0.09	-0.20	0.20
		19	0.00	0.08	-0.16	0.16
		20	0.00	0.05	-0.11	0.11
		21	0.00	0.03	-0.06	0.06
	PAE	1	0.00	0.00	0.00	0.00
		2	19.06	3.15	12.39	25.73
		3	16.44	2.12	11.95	20.93
		4	12.66	1.59	9.29	16.02
		5	8.50	1.17	6.03	10.98
		6	6.22	0.81	4.50	7.94
		7	4.72	0.67	3.30	6.14
		8	3.67	0.57	2.46	4.87
		9	2.67	0.42	1.77	3.56
		10	1.94	0.35	1.19	2.70
		11	1.94	1.73	-1.72	5.61
		12	1.11	0.31	0.47	1.76
		13	0.67	0.24	0.15	1.18
		14	0.55	0.23	0.07	1.04
		15	0.50	0.17	0.15	0.85
		16	0.39	0.15	0.07	0.70
		17	0.22	0.13	-0.05	0.50
		18	0.22	0.09	0.03	0.42
		19	0.17	0.08	0.01	0.33
		20	0.11	0.05	0.00	0.22
		21	0.06	0.03	0.00	0.12
	PI	1	0.00	0.00	0.00	0.00
		2	26.33	3.15	19.66	33.00
		3	23.06	2.12	18.57	27.55
		4	17.50	1.59	14.14	20.86
		5	11.66	1.17	9.19	14.14
		6	8.00	0.81	6.28	9.72
		7	6.56	0.67	5.13	7.98
		8	4.11	0.57	2.91	5.32
		9	3.22	0.42	2.33	4.11
		10	2.06	0.35	1.31	2.81
		11	1.67	1.73	-2.00	5.33
		12	1.22	0.31	0.58	1.87
		13	0.89	0.24	0.37	1.41
		14	0.55	0.23	0.07	1.04
		15	0.39	0.17	0.04	0.74
		16	0.28	0.15	-0.04	0.59
		17	0.17	0.13	-0.11	0.44
		18	0.11	0.09	-0.08	0.31
		19	0.11	0.08	-0.05	0.27
		20	0.11	0.05	0.00	0.22
		21	0.06	0.03	0.00	0.12
	PAE+PI	1	0.00	0.00	0.00	0.00
		2	19.28	3.15	12.61	25.95
		3	16.39	2.12	11.90	20.88
		4	13.11	1.59	9.75	16.47
		5	9.39	1.17	6.91	11.86
		6	6.95	0.81	5.23	8.67
		7	5.44	0.67	4.02	6.87
		8	3.72	0.57	2.52	4.93
		9	2.72	0.42	1.83	3.61
		10	1.67	0.35	0.92	2.42
		11	1.22	1.73	-2.44	4.89
		12	0.83	0.31	0.19	1.48
		13	0.50	0.24	-0.02	1.02
		14	0.39	0.23	-0.10	0.88
		15	0.22	0.17	-0.13	0.57
		16	0.17	0.15	-0.15	0.48
		17	0.17	0.13	-0.11	0.44
		18	0.00	0.09	-0.20	0.20
		19	0.00	0.08	-0.16	0.16
		20	0.00	0.05	-0.11	0.11
		21	0.00	0.03	-0.06	0.06

Supplemental Table 3

Sex	Treatment Group	Distance	Mean	Standard Error	95% Confidence Interval	
					Lower Bound	Upper Bound
Male	Sham	1	0	0	0	0
		2	8.417	1.66	4.8	12.033
		3	11.583	1.64	8.011	15.156
		4	10.861	1.476	7.646	14.076
		5	8.833	1.287	6.03	11.637
		6	7.833	1.28	5.045	10.621
		7	6.027	1.2	3.412	8.641
		8	4.833	1.2	2.219	7.447
		9	3.5	1.05	1.211	5.789
		10	2.722	0.767	1.052	4.393
		11	2.333	0.68	0.851	3.815
		12	1.777	0.495	0.699	2.854
		13	1.027	0.473	-0.003	2.056
		14	0.694	0.371	-0.113	1.502
		15	0.583	0.278	-0.022	1.189
		16	0.527	0.192	0.108	0.945
		17	0.333	0.149	0.008	0.658
		18	0.167	0.075	0.002	0.331
		19	0.167	0.479	-0.877	1.21
		20	-5.55E-17	0.06	-0.131	0.131
		21	2.78E-17	0.03	-0.065	0.065
	PAE	1	0	0	0	0
		2	14.25	2.033	9.821	18.679
		3	15.625	2.008	11.249	20.001
		4	13.875	1.807	9.937	17.813
		5	12	1.576	8.566	15.434
		6	8.875	1.567	5.46	12.29
		7	7.125	1.47	3.923	10.327
		8	5.125	1.469	1.924	8.326
		9	4.125	1.287	1.322	6.928
		10	3.5	0.939	1.454	5.546
		11	2.375	0.833	0.56	4.19
		12	1.875	0.606	0.555	3.195
		13	1.125	0.579	-0.136	2.386
		14	1	0.454	0.011	1.989
		15	0.625	0.34	-0.117	1.367
		16	0.25	0.235	-0.262	0.762
		17	0.125	0.183	-0.273	0.523
		18	-8.33E-17	0.092	-0.201	0.201
		19	-1.67E-16	0.587	-1.278	1.278
		20	5.55E-17	0.073	-0.16	0.16
		21	2.78E-17	0.037	-0.08	0.08
	PI	1	0	0	0	0
		2	15.2	2.033	10.771	19.629
		3	17.2	2.008	12.824	21.576
		4	15.25	1.807	11.312	19.188
		5	13.4	1.576	9.966	16.834
		6	11.725	1.567	8.31	15.14
		7	10	1.47	6.798	13.202
		8	8.625	1.469	5.424	11.826
		9	7.15	1.287	4.347	9.953
		10	5.725	0.939	3.679	7.771
		11	3.975	0.833	2.16	5.79
		12	2.9	0.606	1.58	4.22
		13	2.175	0.579	0.914	3.436
		14	1.775	0.454	0.786	2.764
		15	0.8	0.34	0.058	1.542
		16	0.5	0.235	-0.012	1.012
		17	0.3	0.183	-0.098	0.698
		18	0.2	0.092	-0.001	0.401
		19	2	0.587	0.722	3.278
		20	0	0.073	-0.16	0.16
		21	2.78E-17	0.037	-0.08	0.08
	PAE+PI	1	0	0	0	0
		2	8.65	1.66	5.033	12.267
		3	10.383	1.64	6.811	13.956
		4	9.167	1.476	5.952	12.382
		5	8.317	1.287	5.513	11.12
		6	6.783	1.28	3.995	9.571
		7	6.017	1.2	3.402	8.631
		8	5.283	1.2	2.669	7.897
		9	4.317	1.05	2.028	6.605
		10	2.867	0.767	1.196	4.537
		11	2.05	0.68	0.568	3.532
		12	1.25	0.495	0.172	2.328
		13	1.083	0.473	0.054	2.113
		14	0.833	0.371	0.026	1.641
		15	0.5	0.278	-0.106	1.106
		16	0.333	0.192	-0.085	0.752
		17	0.083	0.149	-0.242	0.408
		18	-5.55E-17	0.075	-0.164	0.164
		19	-5.55E-17	0.479	-1.044	1.044
		20	-5.55E-17	0.06	-0.131	0.131
		21	2.78E-17	0.03	-0.065	0.065

Supplemental Table 4

Sex	Treatment Group	Distance	Mean	Standard Error	95% Confidence Interval	
					Lower Bound	Upper Bound
Female	Sham	1	0	0	0	0
		2	10.222	1.66	6.606	13.839
		3	12.166	1.64	8.593	15.738
		4	11.139	1.476	7.924	14.354
		5	9.499	1.287	6.695	12.303
		6	7.332	1.28	4.544	10.12
		7	4.999	1.2	2.385	7.613
		8	3.86	1.2	1.246	6.474
		9	2.917	1.05	0.628	5.205
		10	2.527	0.767	0.856	4.197
		11	2	0.68	0.518	3.482
		12	1.639	0.495	0.561	2.717
		13	1.167	0.473	0.137	2.196
		14	0.807	0.371	-0.001	1.614
		15	0.417	0.278	-0.189	1.022
		16	0.417	0.192	-0.002	0.835
		17	0.25	0.149	-0.075	0.575
		18	0.193	0.075	0.029	0.358
		19	0.193	0.479	-0.85	1.237
		20	-5.55E-17	0.06	-0.131	0.131
		21	2.78E-17	0.03	-0.065	0.065
	PAE	1	0	0	0	0
		2	11.725	2.033	7.296	16.154
		3	13.3	2.008	8.924	17.676
		4	12.5	1.807	8.562	16.438
		5	12.8	1.576	9.366	16.234
		6	11.4	1.567	7.985	14.815
		7	10.425	1.47	7.223	13.627
		8	8.7	1.469	5.499	11.901
		9	7.275	1.287	4.472	10.078
		10	5.525	0.939	3.479	7.571
		11	4.075	0.833	2.26	5.89
		12	2.575	0.606	1.255	3.895
		13	2.05	0.579	0.789	3.311
		14	1.15	0.454	0.161	2.139
		15	1.025	0.34	0.283	1.767
		16	0.8	0.235	0.288	1.312
		17	0.675	0.183	0.277	1.073
		18	0.45	0.092	0.249	0.651
		19	0.125	0.587	-1.153	1.403
		20	5.55E-17	0.073	-0.16	0.16
		21	2.78E-17	0.037	-0.08	0.08
	PI	1	0	0	0	0
		2	10.875	2.033	6.446	15.304
		3	12.25	2.008	7.874	16.626
		4	11.625	1.807	7.687	15.563
		5	9.65	1.576	6.216	13.084
		6	7.775	1.567	4.36	11.19
		7	6.415	1.47	3.213	9.617
		8	4.915	1.469	1.714	8.116
		9	3.71	1.287	0.907	6.513
		10	3	0.939	0.954	5.046
		11	2.46	0.833	0.645	4.275
		12	1.584	0.606	0.264	2.903
		13	1	0.579	-0.261	2.261
		14	0.415	0.454	-0.574	1.404
		15	0.415	0.34	-0.327	1.157
		16	0.209	0.235	-0.304	0.721
		17	0.125	0.183	-0.273	0.523
		18	-5.55E-17	0.092	-0.201	0.201
		19	-2.78E-16	0.587	-1.278	1.278
		20	5.55E-17	0.073	-0.16	0.16
		21	2.78E-17	0.037	-0.08	0.08
	PAE+PI	1	0	0	0	0
		2	10.943	1.66	7.327	14.56
		3	13.137	1.64	9.564	16.709
		4	13.11	1.476	9.895	16.325
		5	11.999	1.287	9.195	14.803
		6	9.917	1.28	7.129	12.705
		7	8.389	1.2	5.775	11.003
		8	6.277	1.2	3.663	8.891
		9	5.417	1.05	3.128	7.705
		10	4.057	0.767	2.386	5.727
		11	3.193	0.68	1.711	4.675
		12	2.445	0.495	1.367	3.522
		13	1.833	0.473	0.804	2.863
		14	1.533	0.371	0.726	2.341
		15	1.027	0.278	0.421	1.632
		16	0.75	0.192	0.332	1.168
		17	0.527	0.149	0.202	0.852
		18	0.387	0.075	0.222	0.551
		19	0.277	0.479	-0.767	1.32
		20	0.277	0.06	0.146	0.407
		21	0.139	0.03	0.074	0.204
